# Exploring the induction of preproinsulin-specific Foxp3^+^ CD4^+^ Treg cells that inhibit CD8^+^ T cell-mediated autoimmune diabetes by DNA vaccination

**DOI:** 10.1038/srep29419

**Published:** 2016-07-11

**Authors:** Katja Stifter, Cornelia Schuster, Michael Schlosser, Bernhard Otto Boehm, Reinhold Schirmbeck

**Affiliations:** 1 Department of Internal Medicine I, Ulm University Medical Center, Ulm, Germany; 2 Department of Medical Biochemistry and Molecular Biology, Research Group of Predictive Diagnostics, University Medical Centre Greifswald, Karlsburg, Germany; 3 Lee Kong Chian School of Medicine, Nanyang Technological University, 636921, Singapore, Singapore; 4 Imperial College London, London, UK

## Abstract

DNA vaccination is a promising strategy to induce effector T cells but also regulatory Foxp3^+^ CD25^+^ CD4^+^ Treg cells and inhibit autoimmune disorders such as type 1 diabetes. Little is known about the antigen requirements that facilitate priming of Treg cells but not autoreactive effector CD8^+^ T cells. We have shown that the injection of preproinsulin (ppins)-expressing pCI/ppins vector into PD-1- or PD-L1-deficient mice induced K^b^/A12-21-monospecific CD8^+^ T cells and autoimmune diabetes. A pCI/ppinsΔA12-21 vector (lacking the critical K^b^/A12-21 epitope) did not induce autoimmune diabetes but elicited a systemic Foxp3^+^ CD25^+^ Treg cell immunity that suppressed diabetes induction by a subsequent injection of the diabetogenic pCI/ppins. TGF-β expression was significantly enhanced in the Foxp3^+^ CD25^+^ Treg cell population of vaccinated/ppins-primed mice. Ablation of Treg cells in vaccinated/ppins-primed mice by anti-CD25 antibody treatment abolished the protective effect of the vaccine and enabled diabetes induction by pCI/ppins. Adoptive transfer of Treg cells from vaccinated/ppins-primed mice into PD-L1^−/−^ hosts efficiently suppressed diabetes induction by pCI/ppins. We narrowed down the Treg-stimulating domain to a 15-residue ppins76–90 peptide. Vaccine-induced Treg cells thus play a crucial role in the control of *de novo* primed autoreactive effector CD8^+^ T cells in this diabetes model.

Type 1 diabetes mellitus (T1D) is an autoimmune disorder, in which insulin-producing beta cells are destroyed by the cellular immune system^1^. Diabetes development is characterized by progressive infiltration of T cells into the pancreatic islets and consecutive beta cell destruction. Disease in man is triggered by poorly defined antigens and factors that finally result in the breakdown of central and/or peripheral tolerance and activation of autoreactive T cells^2^. There is increasing evidence from patients with T1D that autoreactive CD8^+^ T cells play a crucial role in the development of the disease^3^,^4^,^5^,^6^,^7^. Facing a rise in the incidence of T1D there is thus a clear need for the development of immunotherapies that induce or restore peripheral tolerance and prevent T1D in a controlled and antigen-specific manner^8^,^9^,^10^.

Immune tolerance is regulated by a variety of mechanisms and checkpoints that affect the differentiation of lymphocytes in central lymphoid organs as well as mature lymphocytes in the periphery. Tolerance in the periphery is maintained by modulatory interactions through co-inhibitory ‘programmed death-1′ (PD-1)/‘programmed death-ligand-1′ (PD-L1 or B7-H1) signals^11^,^12^ and/or regulatory Foxp3^+^ CD25^+^ CD4^+^ T cells (Tregs) expressing the transcription factor forkhead box p3 (Foxp3) and the alpha chain of IL-2 receptor (CD25)^13^. Treg cells can be divided into naturally occurring Foxp3^+^ CD25^+^ CD4^+^ Treg cells (nTregs) and induced Treg cells (iTregs) which, upon antigen stimulation, specifically arise from conventional CD4^+^ T cells acquiring CD25 and Foxp3 expression outside of the thymus. Both, nTreg and iTreg cells suppress effector T cell responses through a variety of mechanisms. Treg cells can produce anti-inflammatory cytokines and/or impair antigen presenting cell- (APC) or effector T cell- functions by direct cell-to-cell interactions^13^. Furthermore, the co-inhibitory PD-1/PD-L1 pathway plays a crucial role in the regulation of autoimmune diabetes in *non-obese diabetic* NOD mice^14^,^15^,^16^, diabetes development in man^17^,^18^,^19^,^20^ and, in particular may affect the induction and function of autoantigen-specific Foxp3^+^ CD25^+^ CD4^+^ Treg cells^20^,^21^,^22^.

Animal models have been informative to study autoreactive T cell responses as well as immunotherapies to prevent diabetes development^23^,^24^. DNA vaccination is a promising strategy to induce CD4^+^ Treg cells and treat autoimmune disorders such as type 1 diabetes^25^,^26^. However, little is known about the antigen requirements that facilitate priming of CD4^+^ Treg cells (and inhibit autoimmune diabetes), but do not allow the priming of autoreactive effector CD8^+^ T cells by DNA vaccination. Injection of antigen-expressing vectors preferentially stimulates CD8^+^ T cell responses, because they allow direct antigen expression and MHC class I-restricted epitope presentation by *in vivo* transfected APCs. Furthermore, ‘cross-presentation’ of antigenic material, released from non-professional antigen-expressing APCs (e.g., myocytes) to professional APCs (e.g. DCs) facilitated priming of CD8^+^ T-cell responses^27^. Vector-encoded antigens also stimulate CD4^+^ T cells, indicating that endogenously expressed antigens are efficiently processed for MHC class II presentation^28^. It has been shown that a proinsulin (pins)-expressing DNA vaccine reduced the incidence of diabetes in NOD mice^29^ and the frequency of autoreactive CD8^+^ T cells in patients with T1D^30^. Conditions that promote Th1 to Th2 immunodeviation (e.g. co-expression of the insulin B chain and IL-4) or enhance apoptosis (e.g. by co-expression of glutamic acid decarboxylase and the proapoptotic factor Bax) favor the induction of a protective immunity in NOD mice^31^,^32^. However, there is a narrow ridge between the suppression and/or stimulation of T cell-mediated diabetes by ’self’-antigen expressing DNA vaccines. Diabetes development was accelerated in female and male NOD mice after preproinsulin (ppins)-specific DNA immunization, whereas glutamic acid decarboxylase-specific vector DNA conferred partial protection^33^,^34^. It is largely unknown why certain antigens or antigen domains either stimulate diabetogenic effector T cells or induce immunosuppressive Treg cells. Therefore, strategies that selectively induce antigen-specific Treg cells and suppress autoreactive T cell responses would significantly improve the functionality and safety of T1D vaccines.

The major advantage of DNA-based immunization is the flexibility in the design of vectors and the manipulation of endogenous antigen expression and/or antigen processing/presentation in distinct cellular compartments by molecular engineering. We have established a novel diabetes model in coinhibition-deficient PD-L1^−/−^ (B7-H1^−/−^)^35^ and PD-1^−/−^ mice^36^ to characterize the ppins-specific induction (or prevention) of autoreactive CD8^+^ T cells^37^,^38^. A single injection of ppins-encoding (pCI/ppins) DNA into PD-1- or PD-L1-deficient mice efficiently induced severe autoimmune diabetes^37^. Diabetes developed gender-independent in male and female mice with a median onset of 2–3 weeks post-immunization and a cumulative diabetes incidence of >95% by week 3–5^37^. Epitope recognition of pancreas-infiltrating CD8^+^ T cells isolated from pCI/ppins-immune, diabetic PD-L1^−/−^ and PD-1^−/−^ mice was confined to the K^b^-restricted A_12-21_ epitope at the COOH-terminus of ppins (i.e., the insulin A-chain; Fig. 1a)^37^. A vector-encoded, mutant ppinsΔA12-21 antigen (lacking the critical K^b^/A12-21 epitope) did not induce autoimmune diabetes in PD-L1^−/−^ or PD-1^−/−^ mice^38^. We thus hypothesized that the mutant pCI/ppinsΔA12-21 vector could elicit a prophylactic, Treg cell-mediated immunity in PD-L1^−/−^ and PD-1^−/−^ mice and protect them from autoimmune diabetes induced by a subsequent injection of the diabetogenic pCI/ppins vector. This model allowed us to explore systematically under well-controlled experimental conditions the induction of ppins-specific Treg cells and the suppression of K^b^/A_12-21_-monospecific effector CD8^+^ T cells and autoimmune diabetes.

## Results

### A pCI/ppinsΔA_12-21_ vaccine (lacking the K^b^/A_12-21_ epitope) efficiently suppressed *de novo* induction of autoimmune diabetes by the pCI/ppins vector

**Figure 1:**
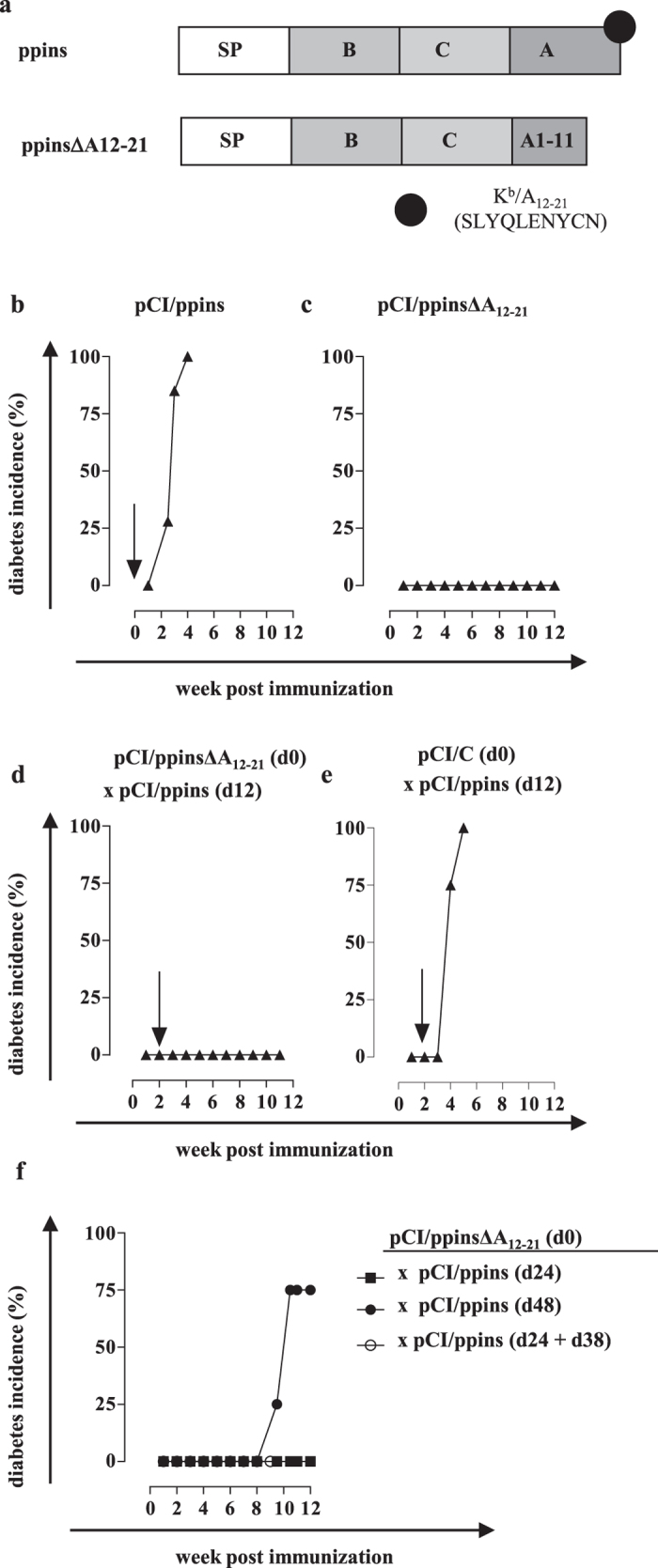
Vaccination of PD-L1^−/−^ mice with pCI/ppins∆A_12-21_ prevents diabetes induction by a subsequent injection of pCI/ppins. (**a**) Map of ppins and pCI/ppinsΔA_12-21_ (lacking the COOH-terminal K^b^-binding epitope A12-21) antigens. The signal peptide (SP), the insulin B- and A-chains, the C-peptide as well as the position and sequence of the K^b^/A12-21 epitope are indicated. (**b–f**) PD-L1^−/−^ mice were either immunized with pCI/ppins (**b**; n = 7) or pCI/ppins∆_12-21_ (**c**; n = 10). Furthermore, groups of mice were either vaccinated with pCI/ppins∆A_12-21_ (**d**; n = 7) or a HBV core expressing pCI/C vector (**e**; n = 3) followed by an injection of the diabetogenic pCI/ppins at day 12 post vaccination. (**f**) PD-L1^−/−^ mice (n = 4 per group) were vaccinated with pCI/ppins∆A_12-21_ followed by an injection of the diabetogenic pCI/ppins at d24 or d48, or by two injections at day 24 and 38 post vaccination. Arrows indicate the injection of the diabetogenic pCI/ppins. Blood glucose values were measured and cumulative diabetes incidences (%) were determined.

Injection of pCI/ppins but not pCI/ppinsΔA_12-21_ DNA (lacking the critical K^b^/A_12-21_ epitope; Fig. 1a) induced autoimmune diabetes in PD-L1^−/−^ and PD-1^−/−^ mice (Fig. 1b,c; Supplementary Fig. S1a, b)^37^,^38^. To investigate whether the modified ppinsΔA_12-21_ antigen induces a prophylactic immunity, we immunized PD-L1^−/−^ or PD-1^−/−^ mice with pCI/ppinsΔA_12-21_ followed by an injection with the diabetogenic pCI/ppins vector at day 12 post vaccination. Interestingly, none of the mice developed autoimmune diabetes (Fig. 1d; Supplementary Fig. S1c). Vaccination of mice with a Hepatitis B Virus (HBV) core antigen-expressing pCI/C vector did not suppress diabetes induction by a subsequent injection of pCI/ppins (Fig. 1e) and, *vice versa*, the injection of pCI/ppinsΔA_12-21_ did not affect the priming of HBV core (K^b^/C_93-100_)-specific CD8^+^ T cells by a subsequent injection (after 12 days) of the pCI/C vector (Supplementary Fig. S2). The immune response induced by the pCI/ppinsΔA_12-21_ vaccine was thus specific for the ppins antigen and suppressed diabetes development by *de novo* activated K^b^/A_12-21_-specific effector CD8^+^ T cells.

The pCI/ppinsΔA_12-21_ vaccine efficiently suppressed diabetes development when the diabetogenic pCI/ppins vector was injected after 12, 24 but not 48 days (Fig. 1d,f). Repeated injections of pCI/ppins at d24 and d38 could not override the protective immunity induced by the initial injection of pCI/ppinsΔA_12-21_ (Fig. 1f). A single injection of the pCI/ppinsΔA_12-21_ vaccine thus induced a potent but temporary immunosuppressive immunity in co-inhibition deficient PD-L1^−/−^ mice.

### PD-L1-independent induction of immunosuppressive responses in wild-type C57BL/6 mice by pCI/ppinsΔA_12-21_

Little is known whether the missing co-inhibitory PD-1/PD-L1 signals in PD-1^−/−^ and PD-L1^−/−^ mice affect the priming of tolerogenic immune responses^21^,^22^. We previously showed that a single injection of pCI/ppins into PD-1/PD-L1-competent C57BL/6 (B6) mice induced IFNγ^+^ K^b^/A_12-21_-monospecific CD8^+^ T cells, but these cells destroyed insulin-producing beta cells only after the injection of anti-PD-L1 antibody^37^. This showed that the priming of autoreactive IFNγ^+^ K^b^/A_12-21_-specific CD8^+^ T cells by pCI/ppins is independent from co-inhibitory PD-1/PD-L1 signals^37^. In contrast, ppinsΔA_12-21_-vaccinated/ppins-primed B6 mice did not develop autoimmune diabetes and anti-PD-L1 antibody treatment failed to induce autoimmune diabetes (Supplementary Fig. S3) providing evidence that the pCI/ppinsΔA_12-21_ vaccine induced a prophylactic immunity in both, co-inhibition-competent B6 and co-inhibition-deficient PD-L1^−/−^ and PD-1^−/−^ mice.

### The pCI/ppinsΔA_12-21_ vaccine induced regulatory Foxp3^+^ CD25^+^ CD4^+^ Treg cells

**Figure 2:**
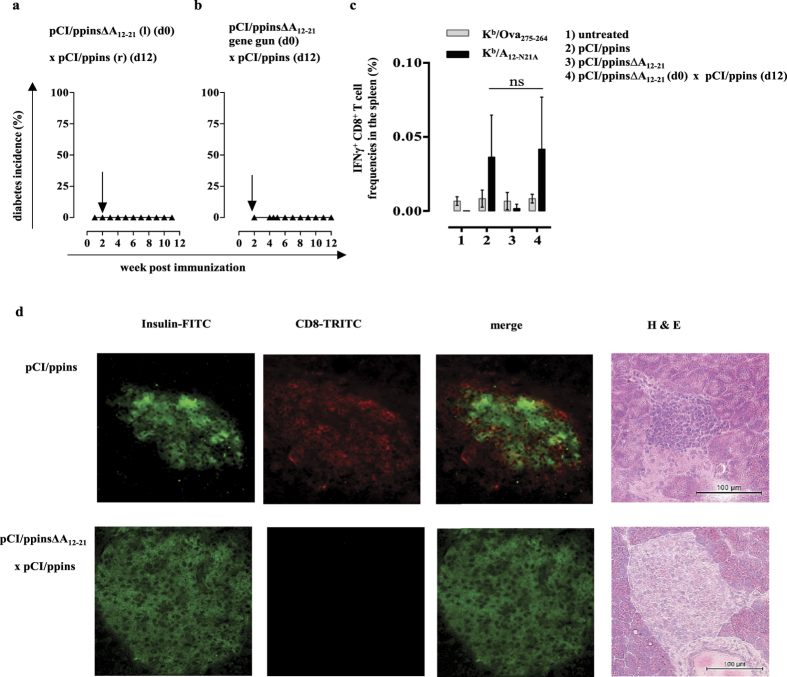
The pCI/ppins∆A_12-21_-induced immune response did not prevent priming of K^b^/A_12-21_-specific CD8^+^ T cells by pCI/ppins. (**a**) The pCI/ppins∆A_12-21_ vaccine was injected into the left (l) and the diabetogenic pCI/ppins (after 12 days) into the right (r) tibialis anterior muscles of PD-L1^−/−^ mice (n = 3). (**b**) Alternatively, the pCI/ppins∆A_12-21_ vaccine was administered intradermally into the abdominal skin with a gene gun followed by an injection of the diabetogenic pCI/ppins vector into both tibialis anterior muscles after 12 days (n = 4). Diabetes development was monitored by regular blood glucose measurements. (**c**) PD-L1^−/−^ mice (n = 3/group) were either left untreated (group 1), or immunized with pCI/ppins (group 2), pCI/ppins∆A_12-21_ (group 3) or pCI/ppins∆A_12-21_ and (after 12 days) pCI/ppins (group 4). Spleen cells were prepared at d12 after the final DNA injection, respectively, and restimulated *ex vivo* with the ppins-specific K^b^/A_12-N21A_ (SLYQLENYCA) peptide^40^. Furthermore, we stimulated spleen cells with a K^b^-binding control peptide (K^b^/Ova_275-264_; SIINFEKL) to ensure that lymphocyte preparations specifically express IFNγ upon ppins-specific stimulation *in vitro*^40^. Frequencies of IFNγ^+^ CD8^+^ T cells were determined by flow cytometry (FCM). The mean % of IFNγ^+^ CD8^+^ T cells in the splenic CD8^+^ T cell population (+SD) of a representative experiment (out of two experiments performed) is shown. Statistically significant differences between the groups 2 and 4 were determined using the unpaired student’s t-test (ns, not significant). (**d**) Insulin expression and CD8^+^ T cell influx into pancreata of representative healthy, pCI/ppinsΔA_12-21_ vaccinated/ppins-primed (lower panel) and diabetic, pCI/ppins-primed PD-L1^−/−^ mice (upper panel) were analysed by histology and hematoxylin/eosin (H&E) staining (see Supplementary Protocols).

In the experiments described above, we injected both, the pCI/ppinsΔA_12-21_ vaccine and the diabetes-inducing pCI/ppins vector into the same *tibialis anterior* muscles. To exclude that the vaccine affected *de novo* priming of K^b^/A_12-21_-specific CD8^+^ T cells at the level of the intramuscular injection site (or in the regional draining lymph nodes), we injected the pCI/ppinsΔA_12-21_ vaccine into the left muscles followed (after 12 days) by an injection of the diabetogenic pCI/ppins vector into the right muscles (Fig. 2a). Alternatively, we administered the pCI/ppinsΔA_12-21_ vaccine intradermally into the shaved abdominal skin using a helium-driven gene gun (see Supplementary Protocols), followed (after 12 days) by an i.m. injection of the diabetogenic pCI/ppins DNA (Fig. 2b). Again, none of these mice developed autoimmune diabetes (Fig. 2a,b) indicating that systemic but not local vaccine-induced responses suppressed *de novo* primed autoreactive effector CD8^+^ T cells.

We detected comparable frequencies of IFNγ^+^ K^b^/A_12-21_-specific CD8^+^ T cells in the spleens of ppins-primed and ppinsΔA_12-21_-vaccinated/ppins-primed PD-L1^−/−^ mice (Fig. 2c; groups 2 and 4) (see Supplementary Protocols). IFNγ^+^ K^b^/A_12-21_-specific CD8^+^ T cells were not detectable in non-immunized or pCI/ppinsΔA_12-21_-vaccinated mice (Fig. 2c; groups 1 and 3). None of the lymphocyte preparations were stimulated by the control K^b^/Ova_257-264_ (SIINFEKL) peptide (Fig. 2c; groups 1–4), confirming the ppins-specificity of the IFNγ response. The pCI/ppinsΔA_12-21_-induced systemic immunity thus did not affect *de novo* priming of autoreactive IFNγ^+^ K^b^/A_12-21_-specific CD8^+^ T cells by pCI/ppins. In contrast, we detected a progressive influx of CD8^+^ T cells into the pancreatic target tissue and beta cell destruction in diabetic, ppins-primed PD-L1^−/−^ mice^38^, but not in healthy, ppinsΔA_12-21_-vaccinated/ppins-primed PD-L1^−/−^ mice (Fig. 2d). These findings suggested that vaccine-induced immune responses impaired trafficking of *de novo* primed K^b^/A_12-21_-specific effector CD8^+^ T cells to the pancreatic target tissue and/or eliminated (or silenced) them in the pancreas^39^. The low affinity A_12-21_ epitope or presentation-optimized peptide variants (i.e., the A_12-N21A_ peptide) inefficiently bound K^b^-molecules and we could not generate epitope-specific K^b^-dimers or tetramers^40^. Therefore, we were not able to track and characterize this critical CD8^+^ T cell population in vaccinated mice.

**Figure 3:**
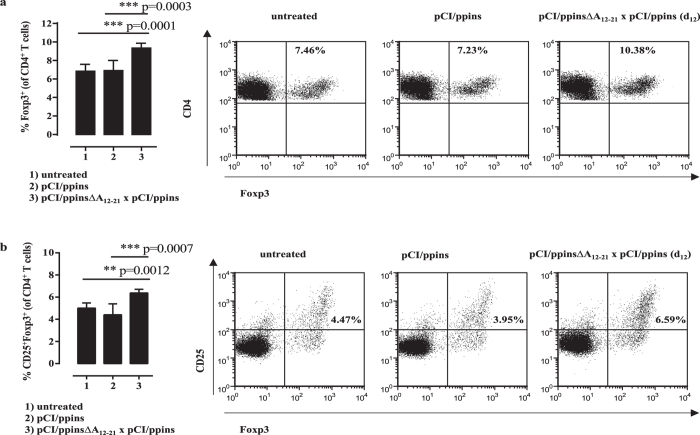
The ppins∆A_12-21_ vaccine induced Foxp3^+^ CD25^+^ Treg cells. Splenic lymphocytes of untreated (group 1, n = 3), pCI/ppins-primed (group 2, n = 3) or ppins∆A_12-21_-vaccinated/ppins-primed (group 3, n = 3) PD-L1^−/−^ mice were isolated 20 days after the injection of pCI/ppins, stained for surface CD4 and CD25 and intracellular Foxp3 expression and analysed by FCM. The actual percentage of Foxp3^+^ (**a**) and Foxp3^+^ CD25^+^ (**b**) Treg cells in gated CD4^+^ T cell populations ± SD of a representative experiment (out of two experiments performed) is shown (left panels). Statistically significant differences between the group 1 and group 3 or group 2 and group 3 were determined using the unpaired student’s t-test. P values of <0.05 (*), <0.01 (**) and <0.001 (***) were considered statistically significant. As example for the underlying flow cytometric analysis, Dot Plots from a representative mouse per group are shown (right panels).

**Figure 4:**
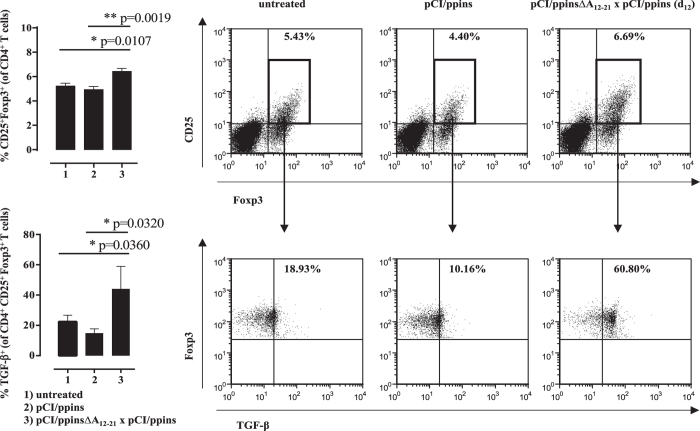
The ppins∆A_12-21_ vaccine specifically induced TGF-β expressing Foxp3^+^ CD25^+^ Treg cells. Splenic lymphocytes of untreated (group 1, n = 3), pCI/ppins-primed (group 2, n = 3) or ppins∆A_12-21_-vaccinated/ppins-primed (group 3, n = 3) PD-L1^−/−^ mice were isolated 21 days after the injection of pCI/ppins. Spleen cells were treated with PMA/ionomycin (see Supplementary Protocols), followed by surface CD4 and CD25 staining and intracellular Foxp3 and TGF-β staining. The actual percentage of Foxp3^+^ CD25^+^ Treg cells in gated CD4^+^ T cell populations ± SD is shown (upper panels). Dot Plots from a representative mouse per group are shown. Boxed areas represent the gates set for analyzing TGF-β expression in Foxp3^+^ CD25^+^ Treg cells (lower panels). Statistically significant differences between the group 1 and group 3 or group 2 and group 3 were determined using the unpaired student’s t-test. P values of <0.05 (*) and <0.01 (**) were considered statistically significant.

Key drivers of the pCI/ppinsΔA_12-21_-induced protective immunity could be regulatory Foxp3^+^ CD25^+^ CD4^+^ Treg cells that eliminate or reprogram autoreactive CD8^+^ T cells^13^. We detected significant higher numbers of Foxp3^+^ and Foxp3^+^ CD25^+^ Treg cells in the splenic CD4^+^ T cell population of ppinsΔA_12-21_-vaccinated/ppins-primed PD-L1^−/−^ mice, than in pCI/ppins-immune or untreated mice (Fig. 3a,b). Furthermore, transforming growth factor beta (TGF-β) expression was augmented selectively in the Foxp3^+^ CD25^+^ Treg cell population of vaccinated/ppins-primed mice after non-specific stimulation with PMA/ionomycin (see Supplementary Protocols; Fig. 4). This indicated that TGF-β producing Treg cells could play a crucial role in the control of pCI/ppins-primed autoreactive effector CD8^+^ T cells and autoimmune diabetes^13^,^41^,^42^.

**Figure 5:**
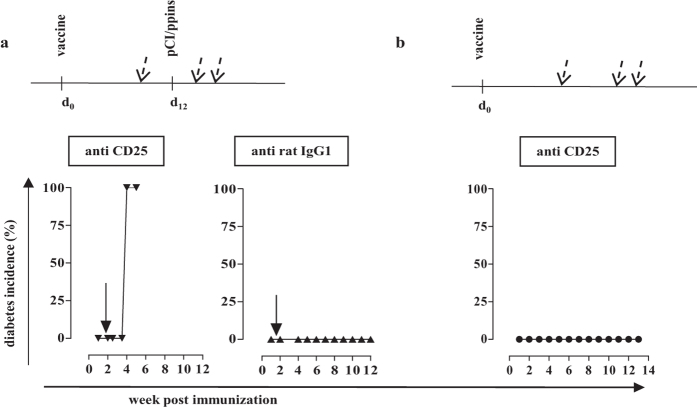
Ablation of Treg cells by anti-CD25 antibody abolished the protective effect of the pCI/ppinsΔA_12-21_-vaccine. (**a**) PD-L1^−/−^ mice (n = 4) were immunized with pCI/ppinsΔA_12-21_ (d0) followed by an injection of pCI/ppins after 12 days. Furthermore, vaccinated/ppins-primed mice were treated three times (at day 3 before and days 3 and 6 after the injection of pCI/ppins; dashed arrows) with 120 μg anti-CD25 mAb PC61 (left panel) or 120 μg rat IgG1 κ isotype control (right panel). (**b**) PD-L1^−/−^ mice (*n* = 4) were vaccinated with pCI/ppinsΔA12-21 and treated at the same days with anti CD25 mAb as described above, but without the injection of the pCI/ppins vector. Arrows indicate the injection of the diabetogenic pCI/ppins DNA at day 12 post vaccination. Blood glucose levels were measured and cumulative diabetes incidences (%) were determined by regular blood glucose measurements.

Most interestingly, acute depletion of Foxp3^+^ CD25^+^ Treg cells by anti-CD25 (PC61) antibody injections (Supplementary Fig. S4), but not isotype antibody injections, into vaccinated/ppins-primed PD-L1^−/−^ mice resulted in diabetes development (Fig. 5a). Similarly, anti-CD25 antibody treatment of vaccinated/ppins-primed PD-1^−/−^ mice resulted in diabetes development (Supplementary Fig. S1d). These findings showed that ablation of Treg cells specifically abolished the protective effect of the pCI/ppinsΔA_12-21_-vaccine and enabled diabetes induction by the diabetogenic pCI/ppins vector. Control experiments showed that depletion of Treg cells by anti-CD25 antibody treatment *per se* did not induce autoimmune diabetes in ppinsΔA_12-21_-immune PD-L1^−/−^ mice (Fig. 5b). Furthermore, anti-CD25 treatment of mice had no measurable impact on the priming of CD8^+^ T cells by DNA-based immunization. Comparable frequencies of IFNγ^+^ K^b^/A_12-21_-specific CD8^+^ T cells were induced in non-treated or anti-CD25 treated vaccinated/ppins-primed mice (Supplementary Fig. S5a,b). Similarly, anti-CD25 antibody treatment did not affect the priming of HBV core/(K^b^/C_93-100_)-specific CD8^+^ T cells by pCI/C (Supplementary Fig. S5c,d).

### Vaccine-induced CD4^+^ Treg cells suppressed diabetes induction in adoptively transferred hosts

**Figure 6:**
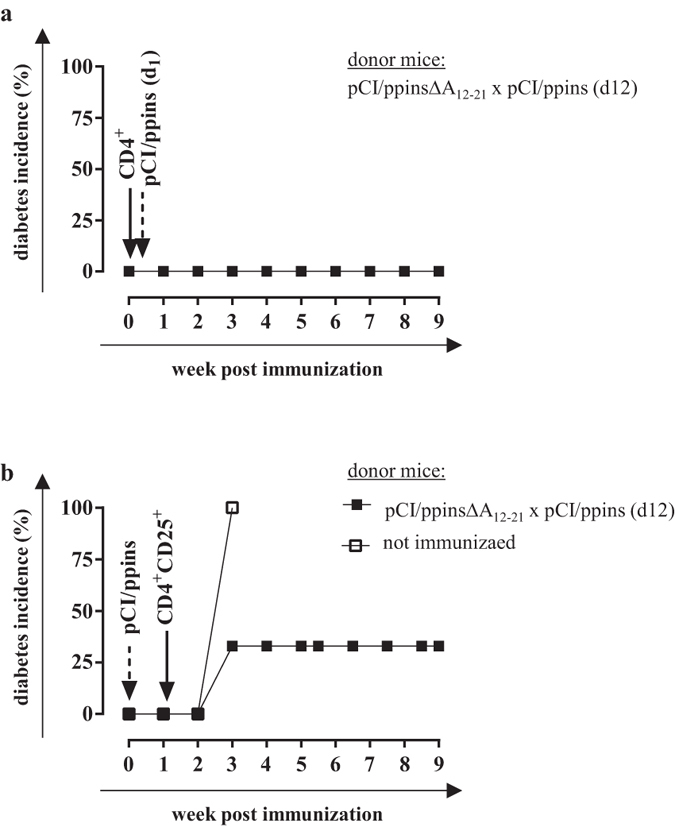
Adoptive transfer of vaccine-induced CD4^+^ T cells into ppins-immune hosts. (**a**) PD-L1^−/−^ mice were immunized with pCI/ppinsΔA_12-21_ (d0) followed by an injection of pCI/ppins after 12 days. After three weeks, splenic CD4^+^ T cells were isolated, magnetically sorted (see Supplementary Protocols) and injected (3 × 10^6^ cells/mouse) intravenously into PD-L1^−/−^ hosts (n = 3) one day prior to the injection of the diabetogenic pCI/ppins. (**b**) CD25^+^ CD4^+^ T cells were sorted from ppinsΔA_12-21_-vaccinated/ppins-primed (see above) or untreated PD-L1^−/−^ mice (see Supplementary Protocols) and injected (3.5 × 10^5^ cells) into PD-L1^−/−^ hosts (n = 3) seven days after the immunization with the diabetogenic pCI/ppins vector. Arrows and dashed arrows indicate the injections of pCI/ppins and purified T cells, respectively. Blood glucose levels were measured and cumulative diabetes incidences (%) were determined by regular blood glucose measurements.

To confirm that vaccine-induced Treg cells are functional *in vivo*, we performed adoptive T cell transfer experiments. CD4^+^ or CD4^+^ CD25^+^ T cells were purified from spleen cell preparations using specific magnetic assisted cell sorting (MACS) kits, respectively (see Supplementary Protocols). Purified CD4^+^ T cells (3 × 10^6^ cells) isolated from vaccinated/ppins-primed PD-L1^−/−^ mice (containing the total pool of CD4^+^ T- and Treg cells) were injected intravenously into PD-L1^−/−^ hosts followed by the injection of the diabetogenic pCI/ppins vector after 1 day. None of these mice developed diabetes (Fig. 6a). Similarly, the transfer of 3.5 × 10^5^ purified CD25^+^ CD4^+^ T cells (containing largely Treg cells, see Fig. 3b) from vaccinated/ppins-primed, but not from non-immunized donor mice into PD-L1^−/−^ hosts one week after the injection of the diabetogenic pCI/ppins DNA efficiently suppressed diabetes induction (Fig. 6b). Vaccine-induced Treg cells thus efficiently suppressed diabetes induction by an ongoing insulin-reactive CD8^+^ T cell response.

### Narrowing down the Treg cell-stimulating ppins domain

**Figure 7:**
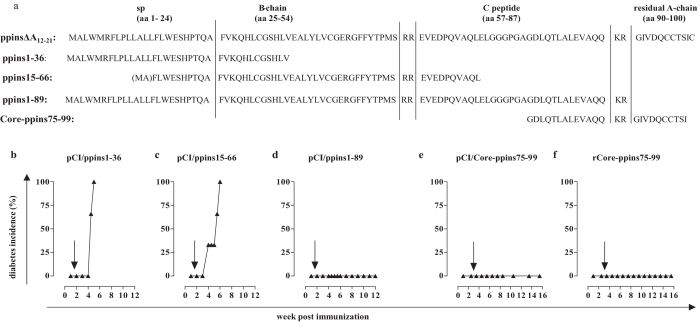
Narrowing down a Treg cell-stimulating domain on the ppinsΔA_12-21_ antigen. (**a**) Aminoacid sequences of ppinsΔA_12-21_, ppins1–36, ppins15–66, ppins1–89 and ppins75–99 are shown. PD-L1^−/−^ mice were vaccinated with pCI/ppins1–36 (**b**) (n = 3), pCI/ppins15–66 (**c**) (n = 3) or pCI/ppins1–89 (**d**) (n = 4) followed by an injection of the diabetogenic pCI/ppins vector after 12 days. (**e,f**) PD-1^−/−^ mice (n = 4) were vaccinated (at day 0 and day 17) with pCI/Core-ppins75–99 encoding a chimeric fusion protein of the HBV Core antigen and ppins77–99 (**e**) or recombinant rCore-ppins75–99 particles adsorbed to alum (**f**) followed by an injection of the diabetogenic pCI/ppins DNA. Arrows indicate the injection of the diabetogenic pCI/ppins DNA. Cumulative diabetes incidences (%) were determined by regular blood glucose measurements.

In H-2^b^ mice, antigen-specific CD4^+^ Treg cells recognize and respond to epitopes presented on the cell surface of APCs by I-A^b^ MHC class II molecules. To narrow down the Treg-stimulating domain(s) on the ppinsΔA_12-21_ antigen, we generated vectors expressing overlapping sequences of this protein (Fig. 7a): a pCI/ppins1–36 vector (encoding the ER-targeting signal peptide up to aa 12 of the B-chain), a pCI/ppins15–66 vector (encoding a sequence from aa 15 of the SP up to aa 10 of the C-chain) and a pCI/ppins1–89 vector (encoding the ER-targeting SP up to the NH_2_-terminus of the A-chain). Injection of pCI/ppins1–89, but not pCI/ppins1–36 or pCI/ppins15–66 vaccines suppressed diabetes development after an injection (at d12 post vaccination) of the diabetogenic pCI/ppins into PD-L1^−/−^ mice (Fig. 7b–d), indicating that the Treg-stimulating domain is localized around ppins67–89. In line with this finding, a 25-residue ppins75–99 fragment (encoding aa 19 of the C-peptide up to aa 10 of the A-chain; C19-A10), expressed as a chimeric fusion antigen with the HBV core antigen (pCI/Core-ppins75–99) (Fig. 7a; Supplementary Fig. S6), induced a prophylactic immunity in vaccinated PD-L1^−/−^ mice (Fig. 7e). Vaccination of PD-L1^−/−^ mice with the pCI/Core-ppins75–99 but not the pCI/C vector (expressing the HBV core without the ppins75–99 insert) efficiently suppressed CD8^+^ T cell-mediated diabetes induction by pCI/ppins (Figs 1e and 7e). Using the particle-forming HBV core antigen as delivery vehicle for the ppins75–99 fragment allowed us to produce recombinant chimeric protein particles^43^ and target directly the exogenous processing pathway for MHC class II presentation and CD4^+^ T cell activation^28^. We produced recombinant rCore-ppins75–99 particles in transiently transfected HEK-293 cells (Supplementary Fig. S6). Vaccination of PD-L1^−/−^ mice with rCore-ppins75–99 particles protected them from pCI/ppins-induced diabetes development (Fig. 7f). Endogenous (DNA) and exogenous (protein) ppins75–99 vaccines thus induced a protective immunity in PD-L1^−/−^ mice.

**Figure 8:**
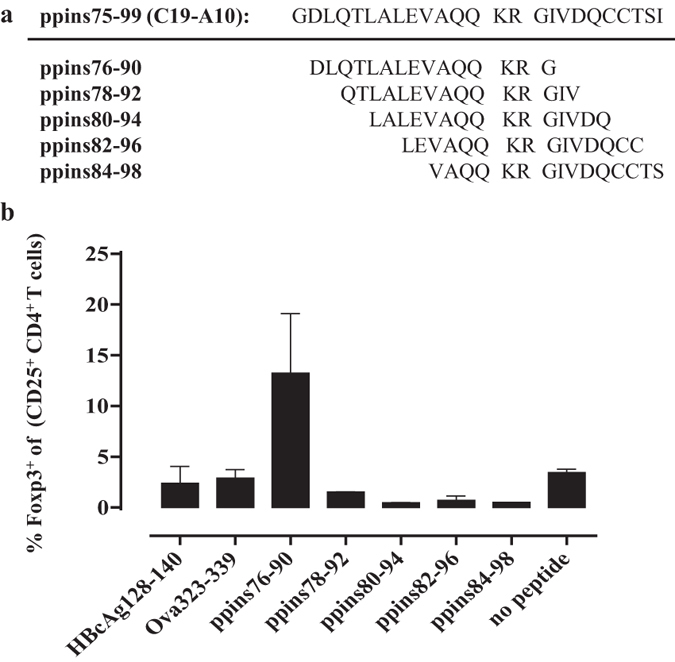
Identification of a Treg-stimulating ppins76–90 peptide. (**a**) Aminoacid sequences of the ppins75–99 (C19-A1) fragment and overlapping 15-residue peptides ppins76–90, ppins78–92, ppins80–94, ppins82–96 and ppins84–98. (**b**) Conventional Foxp3^negative^/eGFP^negative^ CD4^+^ T cells were isolated from spleens of B6-Foxp3^eGFP^ mice using magnetic assisted (MACS) and fluorescence assisted (FACS) cell sorting and stimulated for three days with CD3-depleted autologous splenocytes pulsed with the respective ppins-derived peptides as well as two I-A^b^-binding control peptides (HBcAg_128-140_ and Ova_323-339_). *In vitro* conversion of Foxp3^negative^/eGFP^negative^ CD4^+^ T cells into Foxp3^+^/eGFP^+^ CD25^+^ CD4^+^ T cells was determined by FCM (see Supplementary Protocols). The actual percentages of newly arising Foxp3 expressing CD25^+^ CD4^+^ Treg cells ± SD of a representative experiment (out of two experiments performed) are shown.

Induced iTreg cells specifically arise from conventional CD4^+^ T cells acquiring Foxp3 and CD25 expression upon antigen stimulation^13^. We used B6-Foxp3^eGFP^ mice that co-express the regulatory T cell-specific transcription factor Foxp3 and the enhanced green fluorescent reporter protein (eGFP) primarily in CD4^+^ T cells^44^ to map the Treg-stimulating motif on the ppins75–99 fragment *in vitro*. We isolated conventional Foxp3^negative^/eGFP^negative^ CD4^+^ T cells by magnetic assisted (MACS) and fluorescence assisted (FACS) cell sorting, stimulated them with CD3-depleted autologous splenocytes pulsed with the overlapping 15-residue ppins peptides (Fig. 8a) and determined the actual conversion into Foxp3^+^/eGFP^+^ CD25^+^ CD4^+^ Treg cells (see Supplementary Protocols) (Fig. 8b). The ppins76–90, but not the ppins78–92, ppins80–94, ppins82–96, ppins84–98 and I-A^b^-binding control peptides induced Foxp3^+^/eGFP^+^ CD25^+^ CD4^+^ Treg cells (Fig. 8b). This showed that the ppins76–90 (C19-A1) peptide contains the Treg cell-stimulating motif.

## Discussion

Clinical manifestation of T1D is preceded by the development of autoantibodies to different islet antigens, marking the loss of immunological tolerance to beta cell antigens and an initial destruction of beta cells^45^,^46^. To date attempts to prevent T1D in individuals with HLA genotypes and autoantibodies conferring increased risk of disease were unsuccessful^9^. Therefore, specifically combating the initial autoreactive immune responses before seroconversion by antigen-specific immunotherapies is an attractive approach for preventing or redirecting pathogenic autoimmune reponses^9^,^47^. An optimal approach for the prevention of T1D could include an antigen-specific strategy to induce Foxp3^+^ CD25^+^ CD4^+^ Treg cells^8^. In this study, we used the PD-1/PD-L1 mouse model to explore systematically the induction of ppins-specific Treg cells by DNA vaccination. We showed that a ppinsΔA_12-21_ antigen (lacking the critical K^b^/A12-21 epitope) primed regulatory T cells with a TGF-β^+^ Foxp3^+^ CD25^+^ CD4^+^ signature and efficiently suppressed CD8^+^ T cell-mediated diabetes development by a subsequent injection of the diabetogenic pCI/ppins vector.

Ablation of Treg cells in PD-1^−/−^ and PD-L1^−/−^ mice by anti-CD25 (PC61) antibody injections specifically abolished the protective effect of the vaccines and enabled diabetes induction by the diabetogenic pCI/ppins vector. The clone PC61 has been widely used to examine the function of Foxp3^+^ Treg cells in different murine model systems^48^. The abrogation of the Treg-specific suppressor activity with PC61 mAb could be explained by the actual deletion of CD25^+^ Tregs (Supplementary Fig. S5) and/or by the functional inactivation of these cells^48^. CD25 is expressed on Foxp3^+^ Treg cells but also on activated effector T cells. It is unlikely that anti-CD25 treatment (at the indicated intervals and dose used in this study) has an impact on the priming of CD8^+^ T cell responses by DNA-based immunization. We showed that the treatment of ppinsΔA_12-21_-vaccinated/ppins-primed mice or pCI/C-primed mice with PC61 did not influence *de novo* priming of ppins (K^b^/A_12-21_)- or HBV core (K^b^/C_93-100_)-specific CD8^+^ T cell responses, respectively. Furthermore, the time-course as well as diabetes incidence in anti CD25 treated vaccinated/ppins-primed mice resembled pCI/ppins-induced diabetes in both, PD-L1^−/−^ (Figs 1b and 5a) and PD-1^−/−^ mice (Supplementary Fig. S1a,d). This suggested that vaccine-induced Foxp3^+^ CD25^+^ Treg cells play a major role in silencing the pathogenic effector functions of *de novo* primed IFNγ^+^ K^b^/A_12-21_-specific CD8^+^ T cells in PD-1^−/−^ and PD-L1^−/−^ mice. The mechanism(s) of Treg-mediated suppression of autoreactive K^b^/A_12-21_-specific effector CD8^+^ T cells and diabetes development are unknown. Treg cells suppress effector T cell responses through a variety of mechanisms^13^. Recent studies have defined the cytokine TGF-β as a crucial player in peripheral T cell homeostasis, immune tolerance to self antigens and T cell differentiation during immune responses^41^. In particular, Foxp3^+^ Treg cells utilize TGF-β to suppress immune responses but also to facilitate the conversion of naïve T cells into Treg cells and/or to protect themselves against apoptosis^41^,^42^. We here showed that TGF-β expression was augmented selectively in the Foxp3^+^ CD25^+^ Treg cell population of vaccinated/ppins-primed mice (Fig. 4), providing evidence that TGF-β producing Treg cells could play a crucial role in the suppression of *de novo* primed autoreactive effector CD8^+^ T cells and autoimmune diabetes. Vaccine-induced Treg cells may exert their suppressor activities in the pancreas. It has been shown that DNA-based vaccination with an insulin B-chain-expressing vector significantly reduced the incidence of diabetes in transgenic mice that express the nucleoprotein of LCMV (LCMV-NP) in beta cells and are infected with LCMV. The insulin B-chain vaccination was effective through induction of Treg cells that react with the insulin B chain, secrete IL-4, and locally reduce the activity of LCMV-NP-specific autoreactive CD8^+^ T cells in the pancreatic draining lymph nodes^49^. Furthermore, Treg cells primarily impinge on autoimmune diabetes by eliminating pathogenic T cells inside the islets^39^.

Co-inhibitory PD-1/PD-L1 signals may directly affect induction and function of autoantigen-specific Foxp3^+^ CD25^+^ CD4^+^ Treg cells^20^,^21^,^22^. PD-L1-deficient APCs inefficiently convert conventional CD4^+^ T cells into Foxp3^+^ CD25^+^ iTreg cells, indicating that PD-L1 itself has an impact on iTreg development. Furthermore, culturing iTregs with PD-L1-coated beads enhanced and sustained Foxp3 expression as well as the suppressive function of iTreg cells^21^. We here showed that ppins-specific vaccines elicited functional Treg cell responses that suppressed CD8^+^ T cell-mediated diabetes development in PD-1- or PD-L1-deficient mice. This indicated that ppins-specific Treg cells were efficiently induced in the absence of the PD-1/PD-L1 signaling pathway.

DNA- (endogenous) and protein-based (exogenous) vaccines efficiently induced ppins-specific Treg cells and suppressed diabetes development by a subsequent injection of the diabetogenic pCI/ppins vector (see Fig. 7e,f). Exogenous antigens preferentially stimulate CD4^+^ T cell responses, because they are specifically processed and loaded on MHC class II molecules in the exogenous processing pathway^28^. However, little is known how endogenously expressed antigens stimulate CD4^+^ T cells. Endogenously expressed antigens could be directly processed for MHC class II presentation in alternative intracellular pathways^28^. Furthermore, ‘cross-presentation’ of antigenic material that is released from non-professional antigen-expressing cells (e.g., myocytes) to professional APCs (DCs) may facilitate priming of CD4^+^ Treg cell responses by DNA vaccines. MHC class II-binding peptides are usually 10–20 residues long. We narrowed down the Treg cell stimulating ppins-domain *in vivo* to a 25-residue ppins75–99 (C18-A10) fragment by vectors expressing ppins fragments (Fig. 7) and *in vitro* to a 15-residue ppins76–90 (C19-A1) fragment that specifically stimulated the conversion of conventional Foxp3^negative^/eGFP^negative^ CD4^+^ T cells into Foxp3^+^/eGFP^+^ CD25^+^ CD4^+^ Treg cells (Fig. 8). Interestingly, the ppins75–99 (C18-A10) domain also contained overlapping (nested) immunodominant HLA-DRB*0401-restricted epitopes (i.e., C13-C32, C19-A3 and C22-A5)^50^, indicating that this domain is efficiently processed for MHC class II epitope presentation. Structural features and/or intrinsic expression of ppins designer antigens could affect processing and MHC class I- and class II- epitope presentation^40^,^51^. We previously showed that the expression of mutant ppinsΔA_12-21_ and ppins differed substantially in transiently transfected HEK-293 cells^38^. Both, ppins and ppinsΔA_12-21_ contain the ppins signal peptide (SP) (Fig. 1a) that targets the proteins into the *Endoplasmic Reticulum* (ER), where the SP is removed by ER-resident signal peptidases. However, the expression levels ppinsΔA_12-21_ were weaker than that of ppins and treatment of transfectants with the proteasome inhibitors epoxomicin or lactacystin efficiently restored ppinsΔA_12-21_ levels^38^. In contrast, the expression of ppins in transiently transfected HEK-293 cells was not changed by proteasome inhibitors^40^. This indicated that ppinsΔA_12-21_, but not ppins was efficiently processed by proteasomal degradation. The altered endogenous antigen expression and processing of mutant ppins proteins may thus facilitate MHC class II epitope presentation and Treg cell priming by DNA vaccination.

Vaccines against self-proteins contain a non-predictable risk to induce or stimulate autoreactive T cell responses rather than a protective immunity in individual recipients. Factors like MHC I and II composition or genetic factors, but also antigen expression and processing could influence the priming of immune responses^40^,^51^. We here showed that the ppinsΔA_12-21_ antigen (lacking the dominant K^b^/A_12-21_ epitope) induced Treg cells in PD-L1^−/−^ and PD-1^−/−^ mice. In contrast, the pCI/ppinsΔA_12-21_ vaccine elicited insulin B-chain-specific K^b^/B_22-29_-specific CD8^+^ T cells and autoimmune diabetes in RIP-B7.1 tg mice expressing the co-stimulator molecule B7.1 (CD80) in beta cells^38^. This was unexpected, because the pCI/ppins vector did not induce K^b^/B_22-29_-specific CD8^+^ T cells in RIP-B7.1 tg mice and the pCI/ppinsΔA_12-21_ vector did not induce K^b^/B_22-29_-specific CD8^+^ T cells in PD-L1^−/−^ or PD-1^−/−^ mice^37^,^38^. Deletion of the A_12-21_ sequence may generate a secondary antigen that, in contast to ppins, is efficiently processed for K^b^/B_22-29_-specific epitope presentation. Priming and/or expansion of CD8^+^ T cells specific for this epitope required co-stimulatory ‘help’ from transgenic B7.1-expressing beta cells. Similarly, a proinsulin-expressing pCI/pins vector inefficiently induced late autoimmune diabetes in RIP-B7.1 tg mice^40^. Therefore, RIP-B7.1 tg mice can be used to test ppins antigens if they contain a residual risk to induce autoreactive T cell responses and diabetes. Using this mouse model, we previously showed that the expression of ppins designer antigens in the ER by homologous (SP; Fig. 1a) or heterologous ER targeting signal peptides (e.g., derived from the murine Igκ chain) was crucial to induce K^b^/A_12-21_-specific effector CD8^+^ T cells and autoimmune diabetes, indicating that direct expression and processing of ppins antigens in the ER favors MHC class I presentation of the ‘weak’ K^b^/A12-21 epitope^40^. Designer antigens without these signal sequences that are stable expressed in the cytosol and/or the nucleus, for example, by fusing the ppins sequence COOH-terminally to the green fluorescent protein, did not induce K^b^/A_12-21_-specific effector CD8^+^ T cells and autoimmune diabetes in RIP-B7.1 tg mice^40^. In ongoing experiments, we analyse whether ppins designer antigens that prevent antigen expression, processing and/or presentation in the ER could be a general strategy to induce a prophylactic Treg cell-mediated immunity in mice expressing different MHC haplotypes.

## Methods

### Mice

C57BL/6 (B6) mice (Janvier; Le Genets-St-Isle; France), B6-Foxp3^eGFP^ mice (B6.Cg-Foxp3^tm2(EGFP)Tch^/J; Jackson # 006772), PD-1^−/−^ mice^36^ and PD-L1^−/−^ (B7-H1^−/−^) mice^35^ and were bred and kept under standard pathogen-free conditions in the animal colony of Ulm University (Ulm, Germany). All mouse immunization studies were carried out in strict accordance with the recommendations in the Guide for the Care and Use of Laboratory Animals of the German Federal Animal Protection Law. The protocols were approved by the Committee on the Ethics of Animal Experiments of the University of Ulm (Tierforschungszentrum Ulm, Oberberghof) and the Regierungspräsidium Tübingen (Permit Numbers: 1105 and 1199 to RS). All studies were carried out in accordance with the approved guidelines. Immunizations were performed under short time Isofluran anesthesia, and all efforts were made to minimize suffering. Development of autoimmune diabetes was analysed by regular blood glucose measurements and diagnosed if two consecutive blood glucose values (within 2 days) exceeded 250 mg/dl, i.e. 13.8 mmol/l (Disetronic Freestyle, Sulzbach, Germany).

### Construction of expression vectors

The antigenic sequences of the different ppins antigens were codon-optimized and synthesized by GeneArt (Regensburg, Germany). All constructs were cloned into the pCI vector (cat. no. E1731, Promega, Mannheim, Germany) using the *NheI* and *NotI* restriction sites. Batches of DNA were produced in *E. coli* using the Qiagen Plasmid Mega Kit (cat. no. 12183; Qiagen, Hilden, Germany). Where indicated, antigens were modified with a NH_2_-terminal Strep-tag (st) sequence to purify recombinant proteins from lysates of transiently transfected HEK-293 cells as described previously^52^. HEK-293 cells were used, because they can be transfected with high efficacy (≥90%) using the calcium phosphate method and express high levels of vector-encoded antigens^52^.

### Immunization of mice

Mice were immunized into the tibialis anterior muscles with 100 μg/mouse of plasmid DNA or 10 μg/mouse of recombinant antigens adsorbed to alum (Alhydrogel “85”, Brenntag Biosector, Frederikssund, Denmark). Where indicated, mice were treated with blocking PD-L1 (B7-H1) antibody (clone MIH5, cat. no. 16-5982-85, eBioscience, Frankfurt, Germany) or with anti-CD25 antibody PC61 (ImmunoTools GmbH, Friesoythe, Germany).

### Statistical analysis

Data were analysed using PRISM software (GraphPad, San Diego, CA, USA). The statistical significance of differences in the mean T cell frequencies between groups was determined by the unpaired student’s t-test. A value of (*)p < 0.05 was considered significant (**significant at p < 0.01, ***significant at p < 0.001).

## Additional Information

**How to cite this article**: Stifter, K. *et al*. Exploring the induction of preproinsulin-specific Foxp3^+^ CD4^+^ Treg cells that inhibit CD8^+^ T cell-mediated autoimmune diabetes by DNA vaccination. *Sci. Rep.* **6**, 29419; doi: 10.1038/srep29419 (2016).

## Supplementary Material

Supplementary Information
