# Supplementary material for: Exploring the induction of preproinsulin-specific Foxp3+ CD4+ Treg cells that inhibit CD8+ T cell-mediated autoimmune diabetes by DNA vaccination
Source: Sci Rep. 2016 Jul 11;6:29419. doi: 10.1038/srep29419 (PMC4942695; doi:10.1038/srep29419)
Supplement: Supplementary Information [file srep29419-s1.pdf]

Supplementary Figures

& Supplementary Protocols

Exploring the induction of preproinsulin-specific Foxp3^{+} CD4^{+} Treg cells that inhibit CD8^{+} T cell-mediated autoimmune diabetes by DNA vaccination

Katja Stifter, Cornelia Schuster, Michael Schlosser, Bernhard Otto Boehm and Reinhold Schirmbeck

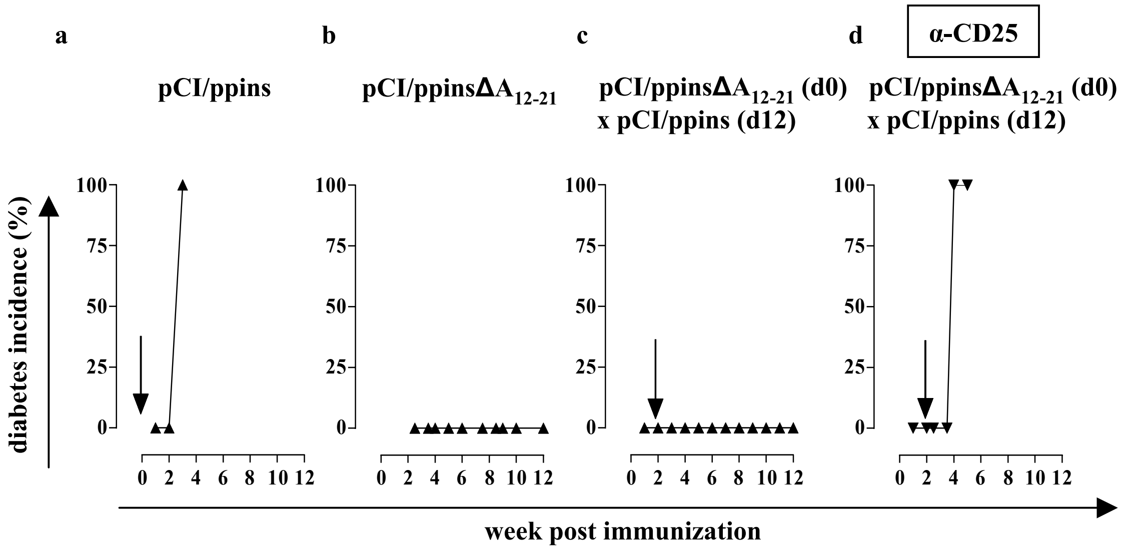

Supplementary Fig. S1

Vaccination with ppins$\Delta A_{12\text{-}21}$ *DNA prevented PD-1*$^{-/-}$ *mice from autoimmune diabetes by a subsequent injection of pCI/ppins DNA.* PD-1$^{-/-}$ mice were either immunized with pCI/ppins (*n*=9, a), pCI/ppins$\Delta_{12\text{-}21}$ (*n*=10, b) or pCI/ppins$\Delta_{12\text{-}21}$ followed (after 12 days) by pCI/ppins (*n*=7, c). A further group (*n*=7, d) of ppins$\Delta_{12\text{-}21}$-vaccinated/ppins-primed mice was additionally treated with anti CD25 mAb (PC61) at day 3 before and days 3 and 6 after the injection of pCI/ppins. Cumulative diabetes incidences (%) were determined by blood glucose measurements. Arrows indicate the injection of pCI/ppins.

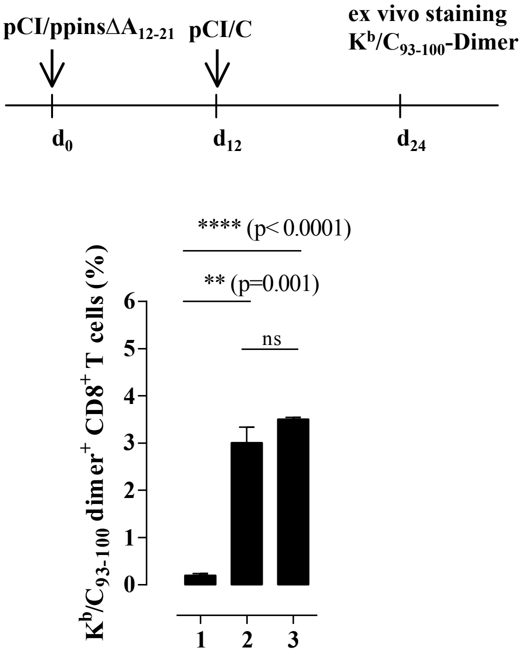

1) untreated

2) pCI/C

3) pCI/ppinsΔA_{12-21} (d_0) + pCI/C (d_{12})

Supplementary Fig. S2

Vaccination with pCI/ppinsΔA_{12-21} does not affect the priming of K^b/C_{93-100} specific CD8+ T cell responses. C57BL/6 mice were immunized with pCI/C (group 2, *n*=5) or vaccinated with pCI/ppinsΔA_{12-21}, followed by a pCI/C injection at day 12 (group 3, *n*=4). On day 12 post pCI/C injection, splenic lymphocytes were prepared from immunized (groups 2 and 3) and untreated (group 1, *n*=4) mice and K^b/C_{93-100}-dimer$^+$ CD8$^+$ T cell frequencies were determined by FCM. Bar graphs show percentages of K^b/C_{93-100}-dimer$^+$ CD8$^+$ T cells ± SD. Statistical significance of differences between the groups was determined using the unpaired student´s t test (ns, not significant).

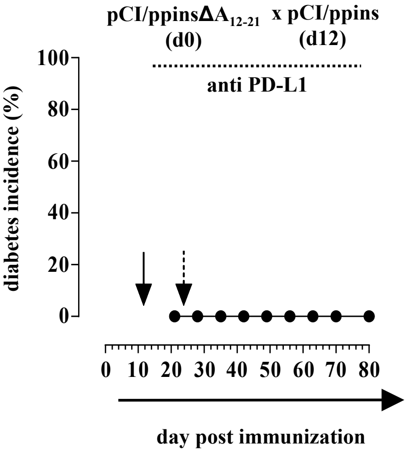

Supplementary Fig. S3

PD-L1-independent induction of immunosuppressive responses in wild-type C57BL/6 (B6) mice by pCI/ppins$\Delta A_{12\text{-}21}$. We previously showed that a single injection of pCI/ppins into PD-1/PD-L1-competent C57BL/6 (B6) mice induced IFNγ^+ $K^b/A_{12\text{-}21}$-monospecific $CD8^+$ T cells, but these cells destroyed insulin-producing beta cells only after the injection of anti-PD-L1 antibody (see reference 37). Here, B6 mice (n=4) were immunized with pCI/ppins$\Delta A_{12\text{-}21}$, followed by the injection of pCI/ppins after 12 days and the injection of anti-PD-L1 mAb at d24. Diabetes development was monitored by regular blood glucose measurements. Arrows and dashed arrows indicate the injections of pCI/ppins and anti-PD-L1 antibody, respectively.

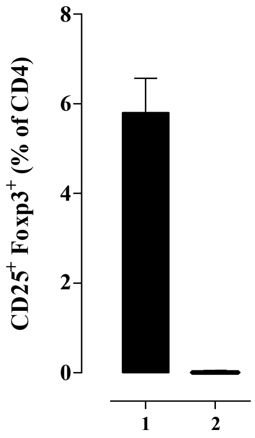

1) pCI/ppins$\Delta A_{12\text{-}21}$ + pCI/ppins
2) pCI/ppins$\Delta A_{12\text{-}21}$ + pCI/ppins (α-CD25)

Supplementary Fig. S4

Treatment of PD-L1$^{-/-}$ mice with anti-CD25 mAb led to acute depletion of CD25^{+} Foxp3^{+} Treg cells. PD-L1$^{-/-}$ mice were vaccinated with pCI/ppins$\Delta A_{12\text{-}21}$, followed by the diabetogenic pCI/ppins injection on day 12. Mice remained either untreated (*n*=3, group 1) or were treated with anti-CD25 mAb (PC61) at day 3 before and days 3 and 6 after the injection of pCI/ppins (*n*=3, group 2). On day 12 after pCI/ppins injection, splenic lymphocytes were prepared from the mice and analysed for CD25^{+} Foxp3^{+} CD4^{+} T cells by FCM.

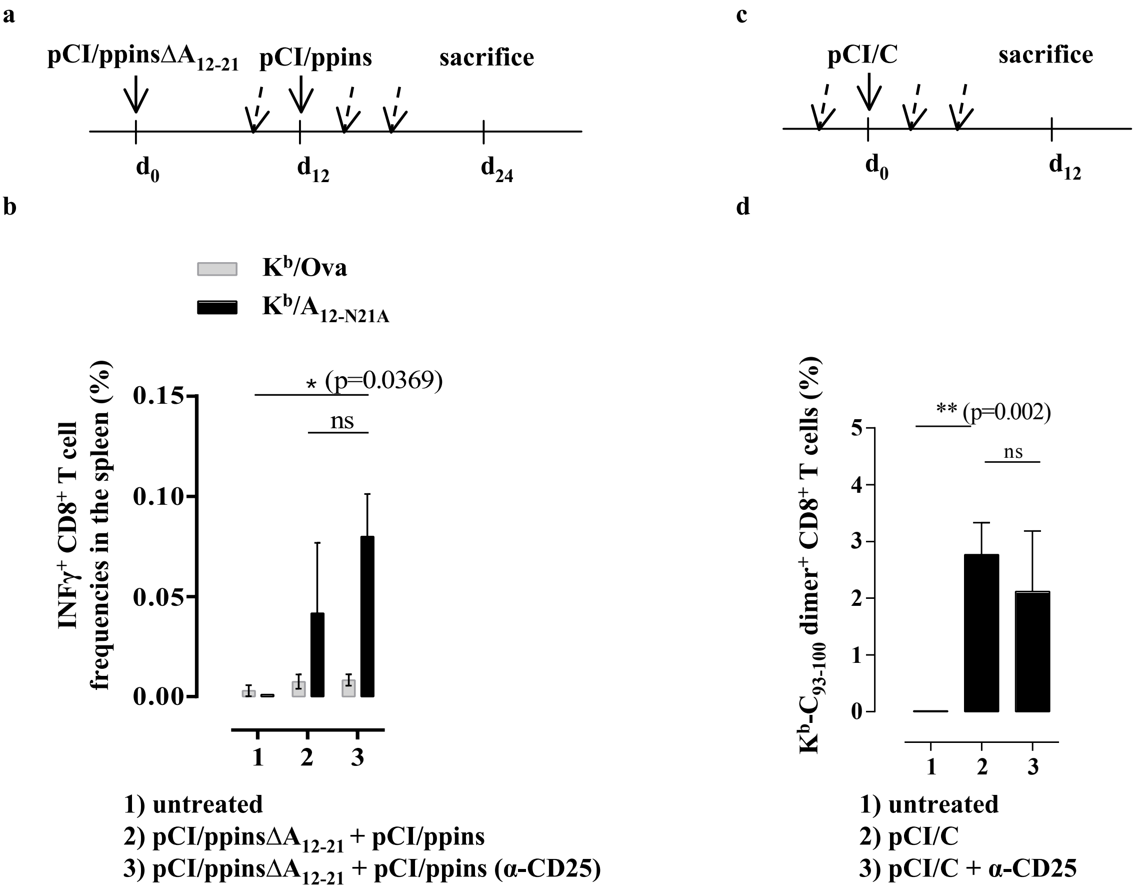

Supplementary Fig. S5

Anti-CD25 antibody treatment did not interfere with the induction of antigen-specific CD8⁺ T cells. (a, c) The general immunization schemes are shown. Dashed arrows indicate the anti-CD25 mAb injections. (b) PD-L1$^{-/-}$ mice were either left untreated (*n*=3, group 1) or vaccinated with pCI/ppinsΔA_{12-21} followed by the injection of pCI/ppins at d12 (*n*=3, groups 2 and 3). Mice in group 3 were additionally treated with anti-CD25 mAb at day 3 before and days 3 and 6 after the injection of pCI/ppins. At day 24 post vaccination, splenic lymphocytes were prepared and *in vitro* re-stimulated with the ppins-$K^b/A_{12\text{-}N21A}$ or a control $K^b/Ova_{257\text{-}264}$ peptide and IFNγ producing $CD8^+$ T cells were determined by FCM. Bar graphs show the percentage of IFNγ$^+$ $CD8^+$ T cells ± SD. (d) C57BL/6 mice were left untreated (group 1, *n*=4), immunized with the HBV core-expressing pCI/C DNA (*n*=3, group 2) or immunized with pCI/C and treated with anti-CD25 mAb (*n*=3, group 3). On day 12 post immunization splenic lymphocytes were prepared and dimer$^+$ $K^b/C_{93\text{-}100}$-specific $CD8^+$ T cell frequencies were determined by FCM. Bar graphs show the percentage of $CD8^+$ T cells specific for the $K^b/C_{93\text{-}100}$-dimers ± SD. Statistical significance of differences between the groups was determined using the unpaired student´s t test (ns, not significant).

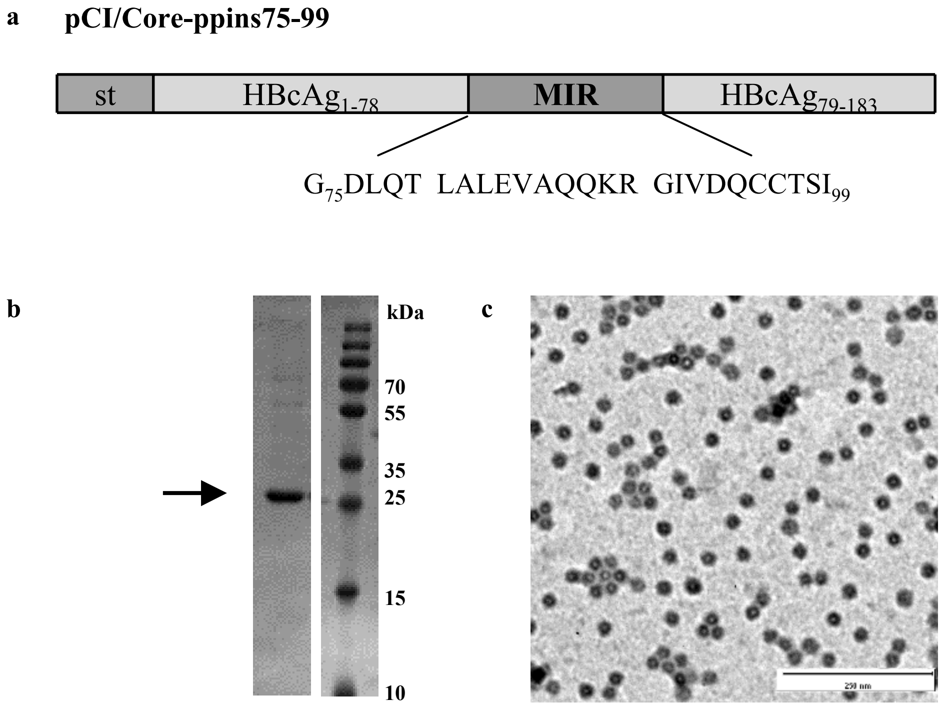

Supplementary Fig. S6

Expression and purification of recombinant rCore-ppins75-99 particles. (a) Map of the pCI/Core-ppins75-99 expression vector. The ppins sequence encoding aa75-99 was inserted into the major immunodominant region (MIR) of the Hepatitis B virus core antigen (HBcAg). A NH_2-terminal StrepTag-sequence (st) was cloned to the fusion construct. (b, c) HEK-293 cells (5 x 10^8 cells) were transiently transfected with pCI/core-ppins75-99. After 48h, cells were lysed and the fusion protein was purified as described in M&M. The recombinant antigen was processed for SDS-PAGE followed by Coomassie Blue staining of the gels. The position of the rCore-ppins75-99 fusion protein is indicated (b). Furthermore, the purified antigen was processed for electron microscopy (magnification 50,000x) (c). The indicated scale bar represents 250nm.

Supplementary Protocols

Exploring the induction of preproinsulin-specific Foxp3$^+$ CD4$^+$ Treg cells that inhibit CD8$^+$ T cell-mediated autoimmune diabetes by DNA vaccination

Katja Stifter[1], Cornelia Schuster[1*], Michael Schlosser[2], Bernhard Otto Boehm[3,4] and Reinhold Schirmbeck[1#]

DNA immunization with the gene gun.
Where indicated small amounts of plasmid DNA (1µg) were intradermally administered into the shaved abdominal skin using a helium driven gene gun (Helios® Gene Gun System, Bio-Rad, Hercules, CA, USA). For intradermal DNA vaccination, plasmid DNA was incubated with spermidine, loaded onto 1µm gold microcarrier particles using $CaCl_2$ (1µg DNA/0.5mg gold) and fixed in a tube (cat. no. 1652441; Bio-Rad) using polyvinylpyrrolidone (PVP). DNA-coated gold particles were injected with a helium pressure of 300 psi [1].

Histology
H&E staining and immunohistochemistry of pancreatic sections was performed as described previously[2,3]. For the staining of insulin and CD8$^+$ T cells the following primary antibodies were used: polyclonal guinea pig anti-insulin serum (cat. no. A0564; Dako, Carpinteria, CA, USA) and rat anti-CD8 (cat. no. MCA2694; Serotec, Oxford, UK). These primary antibodies were detected with the secondary antibodies fluorescein isothiocyanate (FITC)-conjugated anti-guinea pig IgG (cat. no. F-6261; Sigma-Aldrich, Taufkirchen, Germany) and tetramethylrhodamine (TRITC)-conjugated anti-rat IgG (cat. no. T4280; Sigma-Aldrich). Sections were covered and mounted with Cytoseal60 mounting medium (cat. no. 18006, Electron Microscopy Sciences, Hatfield, PA, USA). Finally, the images were captured with an Olympus IX71 fluorescence microscope equipped with a digital camera (C4742, Hamamatsu). Edition of the pictures was performed using ImageJ software (http://rsbweb.nih.gov/if/).

Isolation of T cells and flow cytometric analyses
Mouse splenocytes were isolated as described previously[3]. Cells were washed twice in phosphate-buffered saline containing 1% w/v bovine serum albumin and erythrocytes were removed by a 4 min incubation in lysis buffer (144 mM NH_4Cl, 17 mM Tris, pH 7.2). To detect ppins (K^b/A_{12-21})-specific CD8$^+$ T cell responses, we used the synthetic ppins A-chain derived peptide $K^b/A_{12-N21A}$ and a control $K^b/OVA_{257-264}$ peptide (Thermo Fisher Scientific GmbH, Ulm, Germany). The peptide variant $K^b/A_{12-N21A}$ (containing an alanine exchange for the COOH-terminal asparagine at position A21) was used instead of the K^b/A_{12-21} peptide, because it facilitated *in vitro* expansion of primed CD8$^+$ T cells[4]. Spleen cells (10^6/100 µl) were incubated for 16 h in Ultra Culture medium (cat. no. BE 12-725F, Lonza, Belgium) containing 10 µg/ml of the respective peptides in the presence of 0.5 µg/ml brefeldin A (cat. no. 15870, Sigma-Aldrich). Cells were harvested, surface stained with allophycocyanin (APC)-conjugated anti-CD8 mAb (cat. no. 17-0081-83, BD Biosciences, Heidelberg,

Germany), fixed with 2% paraformaldehyde, resuspended in permeabilization buffer (HBSS, 0.5% BSA, 0.5% saponin, 0.05% sodium azide) and stained with FITC-conjugated anti-IFNγ antibody (cat. no.554411, BD Biosciences). Non-specific binding of antibodies to Fc-receptor was blocked by preincubating cells with mAb 2.4G2 (cat. no. 01241D, BD Biosciences) directed against the FcγRIII/II CD16/CD32 (0.5 μg mAb/10^6 cells/100 μl). Frequencies of $IFN\gamma^+$ $CD8^+$ T cells were determined by flow cytometry (FCM). For the detection of HBV $K^b/C_{93\text{-}100}$ specific $CD8^+$ T cells, spleen cells were stained with FITC-conjugated anti-CD8 mAb (cat. no. 11-0081, eBioscience) and $K^b/C_{93\text{-}100}$ loaded phycoerythrin (PE)-conjugated dimers (BD™Dimer X, cat. no. 552944, BD Biosciences).

To detect Treg cells, spleen cells were surface stained with anti CD4/APC (cat. no. 17-0042, eBioscience) or biotinylated anti-CD4 mAb (cat. no. 13-0042-85, eBioscience, Heidelberg, Germany) and peridinin chlorophyll (PerCp)-conjugated Streptavidin (cat. no. 405213, BioLegend, San Diego, CA, USA), and allophycocyanin (APC)- or PE/Cyanin7 (Cy7)-conjugated anti-CD25 mAbs (cat. no. 17-0251-82 and cat. no. 25-0251-81, eBioscience). For intracellular Foxp3 staining PE-conjugated anti-mouse/rat Foxp3 antibody (cat. no. 12-5773, eBioscience) and the Foxp3/Transcription Factor Staining Buffer Set (cat. no. 00-5523-00, eBioscience) were used, following the manufacturer´s instructions. Where indicated mouse spleen cells (10^6/100 μl) were treated with 25 ng/ml PMA (phorbol 12-myristate 13-acetate, cat. no. P8139, Sigma-Aldrich) and 1 μg/ml ionomycin (cat. no. I0634, Sigma-Aldrich) for one hour at 37°C, followed by a three hour incubation in the presence of BD GolgiStop™ (cat. no. 554724, BD Biosciences). Thereafter, cells were stained for CD4, CD25 and Foxp3 as described above. Additionally, intracellular TGF-β was detected by biotinylated anti-human/mouse TGF-β1 antibody (cat. no. 521705, BioLegend, San Diego, CA, USA) and Streptavidin-FITC (cat. no. 405201, BioLegend). Non-specific binding of antibodies to Fc-receptor was blocked by preincubating cells with mAb 2.4G2 (as described above). Flow cytometry was performed using a FACS LSR II (BD Biosciences) and FCS Express V3 software (DeNovo software, Glendale, CA, USA).

Isolation of T cells and adoptive transfer

For adoptive T cell transfer experiments, $CD4^+$ or $CD4^+$ $CD25^+$ T cells were purified from splenocyte suspensions using the respective magnetic assisted cell sorting (MACS) kit from Miltenyi Biotec (cat. no. 130-104-454 and cat. no. 130-091-041, Miltenyi Biotec, Bergisch Gladbach, Germany), according to the manufacturer´s instructions. After purification, the cells were counted and defined cell numbers ($3x10^6$ $CD4^+$ T cells or $3.5x10^5$ $CD4^+$ $CD25^+$ T cells) in PBS were injected into the tail vain of the acceptor mice.

***In vitro* conversion of naïve $CD4^+$ T cells**

$CD4^+$ T cells were enriched from splenocytes of B6-$Foxp3^{eGFP}$ mice by MACS using the $CD4^+$ T cells isolation kit from Miltenyi Biotec (cat. no. 130-104-454, Miltenyi Biotec). In a second step, $CD4^+$ $Foxp3^{negative}/eGFP^{negative}$ T cells were purified by fluorescence assisted cell sorting (FACS) using a BD FACSAria III cell sorter (BD Biosciences). The sorted cells were then co-cultured with CD3-depleted (CD3ε MicroBead Kit, cat.no. 130-094-973, Miltenyi Biotec) autologous splenocytes (ratio 1:10) in Ultra Culture medium (cat. no. BE 12-725F, Lonza, Belgium) pulsed with 100 μM of the respective ppins-derived peptides or I-A^b-binding control peptides ($HBcAg_{128\text{-}140}$ or $Ova_{323\text{-}339}$). After 3 days, the cells were stained with anti-CD3-PE (cat. no. 100205, BioLegend), anti-CD4-APC (cat.no. 17-0042-82, eBioscience) and PE/Cy7-conjugated anti-CD25 mAb (cat. no. 552880, BD Bioscience) and analysed for induced Foxp3/eGFP expression by FCM.

References

1. Kwissa,M. et al. Efficent vaccination by intradermal or intramuscular inoculation of plasmid DNA expressing hepatitis B surface antigen under desmin promoter/enhancer control. Vaccine **18**, 2337-2344 (2000).

2. Karges,W. *et al.* Induction of autoimmune diabetes through insulin (but not GAD65) DNA vaccination in nonobese diabetic and in RIP-B7.1 mice. *Diabetes* **51**, 3237-3244 (2002).

3. Brosi,H. *et al.* Processing in the endoplasmic reticulum generates an epitope on the insulin A chain that stimulates diabetogenic CD8 T cell responses. *J. Immunol.* **183**, 7187-7195 (2009).

4. Rajasalu,T. *et al.* Deficiency in B7-H1 (PD-L1)/PD-1 coinhibition triggers pancreatic beta cell-destruction by insulin-specific, murine CD8 T cells. *Diabetes* **59**, 1966-1973 (2010).
